# Introducing a Dynamic Workstation in the Office: Insights in Characteristics of Use and Short-Term Changes of Well-Being in a 12 Week Observational Study

**DOI:** 10.3390/ijerph15112501

**Published:** 2018-11-08

**Authors:** Vera Schellewald, Jens Kleinert, Rolf Ellegast

**Affiliations:** 1 Institute for Occupational Safety and Health of the German Social Accident Insurance (IFA), Alte Heerstr. 111, 53757 Sankt Augustin, Germany; Rolf.Ellegast@dguv.de; 2 Institute of Psychology, German Sport University Cologne, Am Sportpark Müngersdorf 6, 50933 Köln, Germany; kleinert@dshs-koeln.de

**Keywords:** short-term changes, well-being, dynamic office workstation, cycling device

## Abstract

The present field study evaluates the use of dynamic workstations (cycling devices) in a real-life office environment. Specific characteristics of use were recorded and possible relationships with short-term changes in well-being were investigated. For a period of 12 weeks, 36 employees were given free access to eight devices. Frequency, duration and speed of use were self-determined but registered objectively for every event of use. Immediately before and after using a cycling device, employees rated their well-being with a modified version of the EZ-scale from Nitsch to assess changes in the short-term. In total, 817 events of use were registered. On each day of the intervention period one of the devices was used. Participants used the devices between one day to all days present at the office, for 21.09 (SD 0.58) to 31.58 (SD 2.19) minutes on average per event of use per day. Comparing the pre- and post-measurements, a significant increase in well-being after using a cycling device was found. Results of a Generalized Estimating Equations (GEE) analysis showed mixed effects for the duration of use, the speed and variation of speed on the probability of reporting positive changes in recovery, calmness and mood. Therefore, using cycling devices in the office might improve short-term well-being.

## 1. Introduction

Sedentary behavior, especially static prolonged seated postures, is considered to be a major health risk factor that is associated with an increase in all-cause mortality [1,2]. Several studies show that the majority of European adults are engaged in sedentary behavior for an average of five hours per day [3,4,5] and that most of this behavior for working adults occurs in the workplace [6]. This behavior might be strongly influenced by being desk-bound and working in a seated position [7,8] and affects a growing amount of employees working in office environments. In addition to becoming increasingly sedentary in nature, the modern work environment is also rapidly changing in structural terms, including greater workloads, increasing time pressure, and decreasing job security due to more contract and part-time work [9]. These factors might result in higher levels of work-related stress, leading to productivity loss and absenteeism or even severe diagnoses like depression [9].

Early on it was assumed that physical activity might have the potential to counterbalance negative effects on mental health [10]. Nowadays the connection between physical activity and mental and physical health is widely established: physical activity has a moderate effect on reducing state and trait anxiety [11] and is associated with a decreased risk of developing clinical depression [12], enhanced subjective well-being [13], the moderation of stress [14], improved mood state [15,16], increased self-esteem [17,18,19,20,21], and quality of life [22]. As a result, many modern occupational health promotion interventions include physical activity in the workplace not only to improve or maintain physical health but also to support well-being [23,24,25].

Recently developed dynamic office workstations (DOWs) are one option to facilitate light physical activity at the workstation. DOWs combine a walking, elliptical pedaling or cycling movement of the legs whilst working at a desk. In general, research has found that DOWs have the potential to reduce workplace sedentary time by about 12 to 29 min per workday [26,27]. Additionally, a range of different DOW devices have been found to increase energy expenditure and heart rate compared to a seated or standing position [28,29,30,31]. In order to facilitate the implementation of DOWs into office environments, lightweight and portable devices were developed that can be stored underneath a desk and shared with colleagues. Scientific evaluations of those portable DOWs in the field are rare. Those available show a high degree of individual variation in the use of the devices regarding the days used (from 3.6% to 70% of the intervention period, time pedalled per active day (9 to 97 min on average per active day) and bouts of use within one event of use (2–19 bouts a 4–16 min) [32,33,34,35,36].

Although the implementation of DOWs as occupational health promotion measures is gaining more attention, there is very little research in the area of systematically evaluating well-being as an outcome of using DOWs. Of the few existing studies on the subject, well-being has been operationalized in very different ways, with studies assessing feelings of stress [37,38,39], activation/deactivation (e.g., arousal, tiredness) [39,40] or motivation [35].

In terms of stress, early work by Edelson and Danoffz [37] showed lower levels of self-reported feelings of stress while using a walking workstation compared to a seated working condition. Similar results are reported by Tudor-Locke et al. [38], where focus groups reported reduced feelings of stress when asked how using treadmills in a field study for an intervention period of 6 months affected their health. Inconclusive results for stress were found from Sliter and Yuan [39], who conducted an experimental study using both a cycling and a walking workstation: the participants experienced significantly lower stress in the walking condition compared to seated (M = −0.57, *p* ≤ 0.001) or standing (M = −0.38, *p* ≤ 0.01) conditions, but found no significant differences for the cycling condition.

Besides stress, feelings of activation or deactivation have also been assessed in some studies. Sliter and Yuan [39] showed that participants experienced significantly higher levels of arousal while using a walking and a cycling workstation compared to passive conditions (*p* ≤ 0.001). Thompson et al. [41] reported a neutral response from participants in answer to the question of whether they feel more tired at the end of the day when using a treadmill station. Although standard deviations were not reported, the authors stated that this question showed the highest disagreement amongst the participants.

Furthermore, motivation-oriented feelings have been examined. Torbeyns et al. [35] assessed, among other things, the concept of work engagement before and after using cycling ergometers in the office for 20 weeks. Their results reported a trend in the intervention group towards an increase in work engagement after the intervention period.

Not only was well-being assessed in different forms, methodological approaches also differed in the reported studies: in some studies the effects of physical activity on psychological well-being were measured directly after using dynamic workstations in laboratory settings [37,39] and others assessed possible effects in the field after the end of an intervention period [35,39,41]. Therefore, research on well-being as an outcome for using dynamic workstations is inconclusive, both in terms of the results and in terms of the methodological approach.

Although field studies reveal great differences in the frequency, duration of using and intensity or speed of using dynamic workstations, these characteristics had not been taken into account by any of the reported studies on well-being. These different characteristics are of special interest in terms of the effects on well-being, because research on physical activity in general shows a dose-response relationship between the amount of activity and the effects on well-being. For instance, in a study of Hassmén et al. [42] participants felt less depression and stress with an exercise frequency of at least two to three times per week. In terms of intensity, a moderate intensity was found to be the most beneficial activity level in terms of achieving positive effects on subjective well-being [43,44]. Regarding the effect of the length of physical activity, study findings are inconclusive, but a longer duration of a single bout of exercise does not seem to be more efficient for subjective well-being than shorter bouts [43,45].

As a promising innovation for health promotion in real-life office environments, this paper aims to extend the knowledge about the use of cycling devices in the field and to examine the relationships between short-term changes in well-being on the one hand and characteristics of use on the other hand. Therefore, our research questions are:

1. How do employees use cycling devices in a real-life office environment?
2. Does using a cycling device in a real-life office environment correspond with short-term changes in well-being?
3. Do different characteristics of use (average duration of use per event, bouts of use within one event of use, speed and consistency of cycling) relate to possible short-term changes in well-being?

## 2. Materials and Methods

### 2.1. Participants

The present study was conducted in a German telecommunication company at a worksite in Cologne. All department heads were informed about the study and asked if their department would be interested in participating by the Health and Safety Department of the company. After two departments declared their interest in participating, an informational event for 42 employees was held to recruit participants in person. Exclusion criteria were a weight of more than 120 kg (equipment weight rating maximum), chronic cardiovascular or musculoskeletal diseases, and having recently undergone surgery. Two employees showed no interest in participating and another two were excluded by the criteria. A sample of 38 participants started the study. Two participants did not use a cycling device throughout the intervention period and were not included in the data analysis. Therefore, the final sample consists of 36 participants (see Table 1 for anthropometric characteristics of the sample).

The majority of participants were highly educated (intermediate school certificate: 7, A-degrees: 2, vocational education/professional training: 14, university degree: 13) and worked an average of 7.7 (SD 1.2) hours per day and 37.1 (SD 6.7) hours per week. Ten of the 36 participants worked an average of 1.4 (SD 0.6) days from home and all, except one, had flexible working hours.

The information about work tasks was self-reported at baseline. According to that, the major part of a workday was screen-based work (5.1, SD 1.6 h), followed by reading and writing tasks (1.4, SD 1.9 h), and telephone calls (1.3, SD 1.1 h). Around one hour on average was spent with formal and informal talking. Participants’ main tasks included administrative, strategic and service tasks for customer and employees.

### 2.2. Procedure

One week before the intervention period began an informational event took place. The health-specific background of integrating Deskbikes into office work, the timeline of the study, and the measurements to be taken were explained. The participants were shown how to use the Deskbike in a comfortable way and according to occupational health and safety instructions. Additionally, the procedure for borrowing a Deskbike and registering the use with chip-carts using the RFID (radio-frequency identification)-technology to register the individual frequency, duration, and speed of use (in revolutions per minute) was explained. All employees were subsequently allowed to briefly test the device. Immediately after the informational event employees that were willing to participate received a chip-cart.

All questionnaires were available as online surveys. An individual code was generated for each participant and provided per email to register for the online surveys. Anthropometric data and additional information about the participants (see Section 2.1) were collected via self-report before the intervention period started. Each time the participants used a Deskbike they were asked to rate their actual well-being immediately before and after the use of the device in a 2-min survey. At the end of each week participants were asked, via another survey of 1 minute, to indicate the number of days that they were present at the office. The day and time each survey was answered was saved automatically by the program used.

### 2.3. Intervention

The study procedure including all interventions, assessments, and measurements was reviewed and approved by the Institutional Review Board of the German Sports University (Nr.: 049/2018). Only data from individuals who gave their informed consent to participate were collected.

The participants’ workplaces were located on two floors, with employees working in so-called cell offices (one room) of two to four people. The company provided eight Deskbikes (Worktrainer, NL) in total. The Deskbike is a portable cycling ergometer, which can be used in combination with height-adjustable desks. The maximum weight of users approved for using the device is 120 kg and it is available in three different sizes. For this study medium-sized devices for a minimum user height of 165 cm were used (www.deskbike.eu).

On each floor four devices were located centrally at so-called “borrowing stations”. All employees included in the study had free access to these stations. Each time they wanted to use it, they had to collect a device from the borrowing station and return it afterwards. The measurement system registering the use of the device was installed at the borrowing station and recorded the participant code, the day and time, the duration and the speed of use (in revolutions per minute). Resistance is infinitely adjustable at the device itself and was not recorded by our measurement systems. For recording the characteristics of use, small boxes which contained a card reader and a mini computer, had to be placed at box holders installed at each Deskbike. Before using a device participants had to select a box, place it at the box holder of the device chosen, and register their use with their chip-card. After using it, the chip-card had to be removed from the device to stop recording data (see Schellewald et al. [36]) and had to be returned to the borrowing station. The frequency, duration and speed of use was self-determined and recorded throughout all 58 working days of the intervention period.

### 2.4. Measures

This study was conducted as an observational study in a real-life office environment. Therefore all measurements registered were not controlled by the researches, but were self-determined by the participants. The measurements used in the present study are calculated based on every time data of a participant was registered by the measurement systems (event). If participants borrowed a device but did not use it (no movement of the pedals), the event was not included in the data analysis.

#### 2.4.1. Characteristics of Using the Deskbike

For the whole intervention period the total number of events of use and the total number of days the devices were used were registered. Further, for each day of the intervention period the number of participants using a device and the number of events at that day were registered. The duration of all events per day was cumulated as an average value per event per day. Additionally, for each week of the intervention period the number of participants being present at the office and using a device (behavioral compliance of the whole study sample per week in %), the total number of events of use and their average duration were calculated.

For each participant individually the total number of days within the intervention period being present at the office was recorded. Then the total number of events of use and the total number of days a device was used throughout the intervention period (absolute activity) were registered. Taking the individual amount of days present at the office into account, the relative activity was calculated by dividing these days present by the number of days a device was used and expressed as a percentage of the days present at the office (relative activity).

*For each event of use* the following characteristics were recorded:

- *The date* and the code of the RFID-chip card were registered to identify participants using the device at that day of the intervention period.
- *The duration of use* was recorded minute by minute and summarized per event.
- *The total number of bouts of use* was calculated as the total of all periods in which complete revolutions of the pedals per minute were recorded per event. A break was identified as a minute of zero rpm-values.
- *Revolutions per minute (rpm)* were recorded by the chip-card every time the pedals completed one revolution. This measurement shows the individual speed of using a Deskbike.
- *Standard Deviation of rpm* expresses how steadily a participant used the Deskbike in terms of the speed of cycling.
- *Rated Perceived Exertion* was assessed with the RPE-scale (Rated Perceived Exertion [46]). Immediately after using a Deskbike the participants were asked to rate the subjective intensity of using it from 5 (no exertion) to 20 (extremely hard) via the online survey. These outcomes were used to subjectively evaluate the intensity of using the Deskbike.

#### 2.4.2. Short-Term Effects on Well-Being

**Figure 1:**
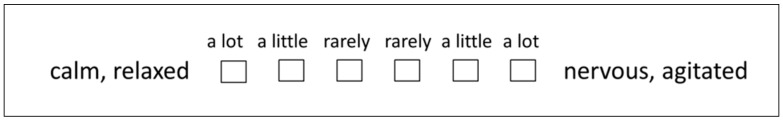
Example of representation of one dimension of the modified EZ-scale.

*Well-being* immediately before and after the use of a Deskbike was assessed by a modified version of the Eigenzustandsskala (EZ-scale) from Nitsch [47]. With the original version, participants characterize their actual state on 40 attributes, mapping motivation and strain on six dimensions. It was designed and validated by Nitsch in 1974 [48] and is widely used for research in occupational health, sports- and clinical psychology [47]. According to our study design, the scale was modified to be feasible for a high number of multiple repeated measurements. This version includes five dimensions, two of which represent motivational state (self-confidence, willingness to perform) and three of which represent stress state (recovery, calm, mood). Each dimension is represented in one bipolar arranged item each containing two semantically similar adjectives at each pole. The two positive adjectives were positioned on the left side and the two negative adjectives on the right side of the six-point answering scale. To avoid a tendency to the middle a six-point answering scale without a mean value was assessed (see Figure 1). For the data analysis the response options were coded from 6 (positive, “a lot”) to 1 (negative, “a lot”). Therefore, a high score expresses positive well-being and a low score corresponds to negative well-being.

### 2.5. Data-Analysis

During the intervention period, data were collected per person and saved in an individual format (person-period). To answer the first research question, total and mean values and standard deviations of different characteristics of use (see Section 2.4.1) were calculated for each event of use (817 in total) collected from 36 participants. As participants differed in their frequency of use of the Deskbikes, between 1 and 88 pre and post-measurements of the dimensions of the modified EZ-scale per participant were registered. Average values per participant were computed for each dimension of the EZ-scale for pre- and post-measurements. Additionally, individual differences from pre to post measurements were calculated by subtracting the pre-values from the post-values and analyzed as indicators of change for each dimension of the modified EZ-scale. For further analysis of possible relationships between characteristics of use and changes of well-being, all events of use including values for the EZ-scale but missing values of characteristics of use were deleted as well as questionnaires which were not answered right after the use of the Deskbike. Therefore 578 events of use from 33 participants remained to be analyzed in inferential statistics. To answer the second research question, a paired *t*-test was conducted comparing the averaged pre- and post- measurements for every dimension of the modified EZ-scale.

As a first step to answer the third research question, an exploration of data was done using graphs and Spearman’s R to detect correlation structures between the characteristics of use and the values for change of well-being as continuous variables. As results showed natural groups in the distribution of data and indicated positive and negative, linear and non-rectilinear correlations, we decided to organize the predictors in categories. The characteristics of use were categorized as illustrated in Table 2, using 25th and 75th percentiles limits. Category 1 includes all values below the 25th percentile limit, category 2 all values from 25th to 75th percentile limit and category 3 all values above the 75th percentile limit. As the duration of use showed a larger variation than the other use characteristics, category 3 included all values from the 75th to the 90th percentile limit and category 4 all values above.

Additionally, the change values for well-being were saved as dichotomous variables with value 1 indicating a positive change (1 to 5 points; i.e., improvement) and value 0 indicating no or a negative change (0 to −5 points; i.e., no improvement). Therefore, the results can be interpreted regarding the general potential of using Deskbikes to improve well-being in short-term.

The analysis of possible relations between the characteristics of use and changes in short-term well-being was done using logistic generalized estimating equations (GEE) regression models with an exchangeable correlation structure. As multicollinearity existed between the predictors, they were computed separately for each predictor and each outcome.

GEE models account for the correlation of repeated measurements on one subject over time [49] and therefore the between-subject and within-subject effects were assumed in the data collected in the present study. All models were adjusted for age, gender and relative activity. Age was being categorized as following: 1 = younger than 37 years, 2 = between 37 and 49 years, 3 = between 50 and 55, 4 = older than 55). The relative activity per participant was divided by less and more than 50% relative activity in two categories.

## 3. Results

### 3.1. Characteristics of Use

**Figure 2:**
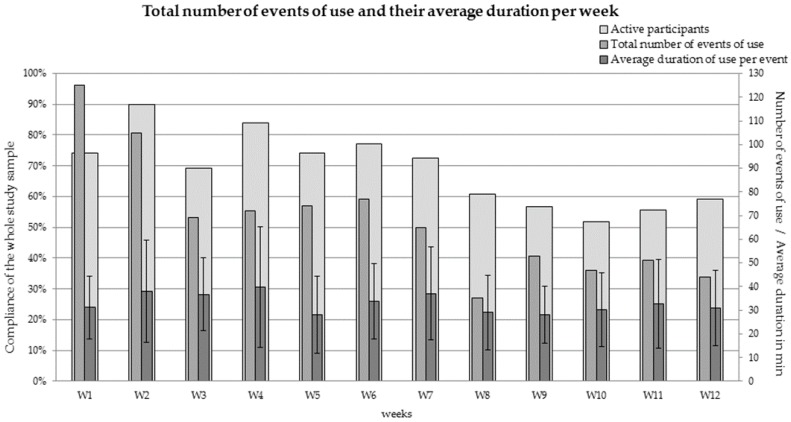
Total number of events of use and their average duration per event per week of the intervention period (*n* = 36).

Throughout the intervention period of 58 days, a total of 817 events of use were registered from all devices. On each day one of the DOWs was used by at least two up to a maximum of 19 participants. The lowest number of events per day was registered with 3 events with an average duration of 21.09 (SD 0.58) minutes per event, the highest number of events per day was registered with 31 events with an average duration of 31.58 (SD 2.19) minutes per event. Half of the participants (*n* = 17) were active on less than 50% of their days present and almost half (*n* = 16) were active on more than 50% of their days present. The total number of events of use per week and their average duration are shown in Figure 2, together with the percentage of all participants being present at the office and using the devices (behavioral compliance of the whole study sample per week; *n* = 36).

Regarding the percentage of participants being present at the office and using the Deskbikes there was a decline from 79.4% (SD 9.4) for the first 4 weeks to 71.1% (SD 7.1) from week 5 to 8 to 55.8% (SD 3.1) for the last 4 weeks. For the first two weeks over 100 events of use were registered per week. For the following two weeks around 70 events per week were registered. This number almost remained until week 8, where the total number of events of use dropped to 35. For the last four weeks a minimum of 44 events (week 8) and a maximum of 53 events (week 9) was registered. The average duration of use for each of these events declined slighty from 35.8 min (SD 19.3) for the first 4 weeks to 32.3 min (SD 17.3) for the middle third of the intervention period. This value remained for the last four weeks (30.4 min, SD 15.6 min) although the number of participants using the devices declined. Over the intervention period of 12 weeks, fewer participants used the Deskbikes in their time present at the office per week. Although the number of events of use per week declined, too, their average duration remained almost the same.

Minimum and maximum values of objective characteristics of use per event (817 in total) for 36 participants can be seen in Table 3.

For 578 events of use, 33 participants rated their subjective percieved exertion after using the cycling devices (see Section 2.5. Data-Analysis). The intensity of all events of use was rated by the study sample with an average of 10.9 (SD 1.7) on the RPE-scale, which correlates to a perception of “fairly light”. The lowest average value of subjective intensity for one participant was 8.4 (SD 1.5; “very light”), the highest one was 13.2 (SD 1.5; “somewhat hard”). In total, 339 events of use were rated with a subjective intensity between 7 and 11 (no effort to very light intensity), 237 events were rated with an intensity between 12 and 15 (moderate to vigorous intensity) and 2 events reached a RPE score of 17 (very high intensity). The greatest difference registered for one participant was found with the lowest rating of 9 (very easy) after using a device for 61 min with an average speed of 32 rpm (SD 12.6 rpm), and the highest of 17 (very intense) after using the device for 19 min with an average speed of 35 rpm (SD 6.1). A total of 17 out of 33 participants showed a range of 4 or more points difference between their individual RPE-scores for separate events of use.

### 3.2. Short-Term Changes in Well-Being

Mean values for the dimensions of the EZ-scale showed large intra- and interindividual differences. The greatest individual difference assessed showed a positive change from a pre-measurement of 1 to a post-measurement of 6 points for the dimension of mood. The greatest negative change was assessed with a pre-measurement of 6 points to a post-measurement of 2 points for the dimension recovery. Although group mean values for the five dimensions of the modified EZ-scale were quite high before the use of a Deskbike, all values increased slightly right after the use of a Deskbike. The results of the paired *t*-test show significant changes for all dimensions of the scale except for recovery. The effect sizes for all dimensions are small [48] (Table 4).

### 3.3. Relationships of Different Characteristics of Use with Short-Term Changes in Well-Being

For inference statistics, logistic GEE models were conducted with categorical variables of characteristics of use as predictors and dichotomous change values for each dimension as outcomes. As the outcome values were characterized as indicators for a trend in change (positive or no/negative change) the regression analyses are modelling the probability of the outcome indicating a positive change. Results of all separate analyses include age, gender and relative activity and values are summarized in Table 5. For the characteristics of use, the category representing the lowest values was used as the reference category. Results showed some associations between age and gender and the probability of reporting a change in the different dimensions of well-being. But as the present paper focuses on possible relationships between characteristics of use and short-term changes in well-being, the associations of age and gender are not further discussed. The relative activity had no significant effect on the probability of reporting positive changes of the dimensions of well-being.

Results of the GEE analyses indicate that three of the five dimensions of well-being were affected by three characteristics of use registered by the measurement systems. The duration of use per event influenced the odds of reporting a positive change in recovery. Events of use lasting 36.13 to 51.83 min showed significant higher odds (OR 2.22) of improving the outcomes in this dimension than events of use with a duration of less than 22.1 min. Additionally the speed of moving the pedals showed a significant effect on the probability to report a positive change in the outcomes of the dimensions recovery and mood. Using the DOWs with a speed of 35.50 to 40.40 rounds per minute has 1.60 higher odds of improving the outcomes for recovery than using them with a speed lower than 35.35 rpm. Using the DOWs with a speed of 35.50 to 40.40 rpm showed 0.50 lower odds of rating the dimension mood higher directly after the use than using them with a speed less than 35.50 rounds per minute. Additionally, the consistency of moving the pedals had an impact on the dimension recovery, mood and calm. Moving the pedals with a variation of more than 10.38 rpm showed a 0.52 times lower probability of feeling more recovered after using the DOWs than moving them with a variation of less than 5.44 rpm. Otherwise, for the dimension calm a variation of rpm of more than 10.38 rpm while using the devices showed 1.82 higher odds of reporting improvements than a variation of less than 5.44 rpm. For the dimension mood a variation between 5.44 to 10.38 rpm while using the DOWs showed 1.61 higher odds that participants report a positive change in the outcomes than a variation of less than 5.44 rpm. The number of use bouts within an event of use had no significant effect on the probability of reporting positive changes of the dimensions of well-being in short-term.

## 4. Discussion

This study evaluated the use of portable dynamic workstations called “Deskbikes” in a real life office environment. Specifically, we took a closer look at different characteristics of use of the devices (duration of use, number of use bouts, speed and consistency of using) and examined short-term effects on well-being. As indicated by the main results, the devices were used regularly by most of the participants. Furthermore, use of the devices led to an increase in well-being in the short-term in general. Three characteristics of use showed significant and mixed effects on the probability of perceiving a positive change in three of the dimensions of well-being (recovery, calm, mood) in the short-term.

Regarding the measurements of characteristics of use in this study, participants showed a similar behavior compared to other studies in the field. Half of the participants used the Deskbikes for more than 50% of their time present at the office, which relates to using a device every second day or either on a daily basis. On the other hand, 16 participants used the devices on less than 50% of their days present. Average values for the behavioral compliance of participants to use dynamic workstations in the office show large differences between study populations as well: participants of Carr et al. [32,33,34] used under-desk pedalling machines between 37% and 70% of the intervention period. Schellewald et al. [36] compared two different kinds of DOWs (activeLife Trainer and Deskbike) in the field, where the Deskbike was used on 3.6% to 86.7% of all days present at the office and the activeLife Trainer on 3.6% to 50% of all days present.

Regarding the overall behavioral compliance of the participants over the study period, there was a slight decline from the first third to the second third of the intervention period. For the first eight weeks, behavioral compliance could be rated as moderate to high with around 75% of all participants being present using the devices in that time. In the last four weeks, the percentage of active participants declined for almost 16% and the total number of events of used dropped to a maximum of 53. We can assume that the higher percentage of participants being active and the higher total number of events of use in the first weeks were a result of the novelty effect of the intervention which did not hold on for the whole study period. Participants maintaining their characteristics of use over the time of the intervention study were probably already more interested in being physically active and increasing their health status in general. This is a very common issue in the field of health intervention measurements. To increase the appeal of using dynamic workstations and keep employees motivated, the introduction of small competitions for groups of employees and additional information about the positive effects of physical activity in general might be effective [50].

As participants of the present study showed a large variety of days present at the office and of total number of events of use, we decided to focus the results on every event of use instead of using average values of use in min per active day for the whole study sample. For the comparison of our results with other studies, however, we have calculated the following exemplary values: participants were present on 32.2 days on average and used the devices on 15.8 days (48.9% of all days present; every second day) for an average of 50.1 (SD 13.4) minutes per active day. These results are similar to the findings of other studies and show even higher durations of use: Carr et al. reported an average duration of use of elliptical under-desk machines of 31.3 [33] and 50 [34] minutes per day pedaled. Torbeyns et al. [35] reported that participants used height-adjustable bike desks for an average of 98 min per week for an intervention period of 20 weeks, which corresponds to using them for 49 min twice a week. Schellewald et al. [36] showed a use of Deskbikes of 49.9 min on average per active day. Participants of the present study intuitively chose a duration of use comparable to the durations registered in those studies as well. Considering that half of all participants used the Deskbikes on more than 50% of their days present, their use of the devices might even contribute to levels of daily light physical activity.

As a record of intensity of use we used the subjective Rating of Perceived Exertion (RPE) on the Borg scale from 6 to 20 [46]. On the one hand, participants scored the intensity of using the Deskbikes between 8.4 (SD 1.5) and 13.2 (SD 1.5) on average for all their events of use, which correlates to light and moderate physical activity intensity according to the ACSM guidelines [51]. Considering that 237 of 578 events of use were rated with an intensity between 12 and 15, nearly half of all times the devices were used with an intensity in the target range for moderate physical exercise [52,53]. On the other hand, the ratings could be interpreted as the perception of the demand of combining the physical activity and desk work. Some of the individual differences registered might support this suggestion, as they showed large variations in between events of use of one person without a change of objective measurements of use like duration or speed of using. Participants might adapt the intensity of use to the type of task they perform during the use to feel comfortable using them and working at the desk at the same time. Therefore using the devices with a health-promoting intensity and working at the same time might not be possible for all office tasks.

Results also show that most of the participants used the Deskbikes regularly and for uninterrupted periods. From all 817 events of use, 583 consisted of one single bout of use and just a few included breaks within. In comparison, the participants of Carr et al. [34] used seated elliptical machines (activeLife Trainer) for an average of 18.6 bouts of use which lasted an average of 4.4 min. Each of the participants was assigned a device on their own, stored underneath their desk. This form of access might explain the different outcomes, as the participants in the present study had to share the Deskbikes with their colleagues. It could be that the system of borrowing devices led to longer bouts of use before returning them. Interrupting the consistency of moving the pedals within one event of use may be a result of changing to tasks that require more or less concentration or precision. Depending on the task, it may be easier or more difficult to integrate the movement of the legs, solving the task and maintaining a good performance (e.g., taking a phone call, copying a text or using the mouse) [54,55,56]. This task-dependence could also explain our findings of a maximum standard deviation of 24.5 rpm, resulting from different speeds of moving while solving changing tasks with different demands. Furthermore, the highest assessment of speed of pedaling with 46.4 rpm on average per use bout is far lower than the measurements of Carr et al. [34], which reported an average of 59 rpm while using the activeLife Trainer. This may be explained by the different leg motion that occurs from the two types of dynamic workstations: while the activeLife Trainer requires a more horizontal movement, using the Deskbike results in a more vertical leg motion. As with cycling, aiming for a higher cadence on the Deskbike might lead to a higher subjective and objective physical exertion [57] that could interfere with office work as well.

In our study the concept of well-being is defined as an individual’s psychic state (“Eigenzustand”; EZ) expressed by experiences of stress, recovery and calm on the one hand and motivation and mood on the other hand. Assessed by the EZ-scale, the use of the Deskbike is associated with a small but significant increase in well-being in the short-term in general. These pre-post-changes are especially remarkable as the initial values of all dimensions of the EZ-scale (recovery, self-confidence, calm, mood and willingness to perform) were already quite high immediately before the Deskbike was used. However, using a Deskbike seems to have the potential to increase an already positive rating of well-being even more. On the one hand, these positive pre-values might reflect a positive work atmosphere. Alternatively, they could be indicative of an already existing motivation to use the device. Specifically, it could be possible that participants use the devices when they already are in a good mood. This suggestion is underlined by scientific findings that negative moods more often undermine the motivation to be physical active [11,58]. Considering that the highest increases in well-being are expected in cases where a poor state of mind is reported before exercising [59], the employees should be trained to specifically use the devices when feeling down or stressed in order to benefit even more from using them.

The pre-post-changes have different effect sizes depending on the different dimensions of well-being. The strongest changes were found for the EZ-dimensions “mood” (0.34) and “willingness to perform” (0.40). These results reflect the particular potential of physical activity to enhance the motivational state (i.e., willingness to perform) and therefore positively influence productivity at the workplace [60,61,62]. Although the results for the dimension “recovery” did not show a significant effect, using the DOWs might still have the potential to reduce symptoms of stress. Experiencing stressful events at the workplace might lead to a decrease in mood [9]. But as the outcomes for the dimension mood showed a positive increase after using the Deskbikes, this could be interpreted as a sign of a positive effect on the perception of stress. Additionally, participants might rather feel more activated than recovered as a result of being physically active.

Stress reduction and motivation enhancement are especially important given the high amount of stress-related problems at work: Around 14% of all workers suffering from a work-related health problem which is associated with stress, anxiety or depression [63,64], accounting for “a significant number of days lost per year” [64] and annual costs to employers in Europe of 272 billion [65]. Therefore the use of cycling devices might enhance the motivation of employees and support a positive work atmosphere.

Results of the GEE models show that three characteristics of use might relate to the perception of change in three dimensions of the EZ-scale. Although the comparison of pre- and post-measurements for the dimension “recovery” showed no significant effect, the duration of use per event, the speed and the consistency of moving the pedals seemed to have an effect on the likelihood of feeling more recovered after using the Deskbike than before. Using the cycling device for a duration of 36 to 52 min had a higher probability of perceiving a positive change compared to using them less than 22.1 min. Otherwise using the devices longer seemed not to be more beneficial for the perception of recovery (*p* 0.09) compared to a short duration. Similar to these effects, there was a significant effect of a moderate speed of moving the pedals (35 to 40 rpm) on the probability of reporting an increase in the values for recovery compared to using the DOWs with a lower speed. Additionally, the variation of speed showed an increase of the probability to report improvements for the category including moderate values (OR 1.34), but 0.52 times lower odds for a variation higher than 10 rpm with a significant effect compared to the lowest values of beneath 5 rpm. These findings might suggest that the DOWs should be used at least for a moderate duration with a moderate speed and variation to feel more recovered than before using them. Otherwise an increase in duration, speed and variation might not have additional effects or even lower the probability of a positive change. These results are quite understandable, if one considers that especially light to moderate physical activity can serve as an active way to recover physically and mentally even in short-term: blood circulation of the muscles and the brain increases and the general oxygen supply of the blood is improved, which in turn improves the ability to concentrate, lowers the level of stress hormones and physical and mental perception of tension states are reduced [66,67,68]. In line with that, higher physical exertion (induced by a longer duration, higher intensity) can lead to physical and mental overload, which correlates to the present results for lower odds of reporting positive outcomes for the dimension recovery for the categories including the highest values for duration, speed and variation of speed.

Opposite to the dimension recovery, a high variation in the speed of moving showed a significant effect for the probability of improving the outcomes in calm (OR 1.82) compared to a low variation. A low variation in the speed of moving indicates a rather continuous movement of the pedals. This might be related to situations where employees carried out monotonous repetitive activities or had to concentrate on tasks where changing the speed of moving might interfere with solving the task. Additionally, solving tasks which require a high degree of concentration will not likely contribute to make employees feel more relaxed. Instead, a moderate to high variation might be an indicator for employees completing all different kinds of tasks which they are more or less familiar with. In this situation, using the Deskbike might contribute to a positive change in the outcomes for the dimension calm, as it may be perceived as a kind of distraction where the thoughts do not have to be focused. On the other hand, repetitive and rhythmic movement, i.e., when swimming, walking, rowing or cycling is likely to elicitate the relaxation response, a state of mental and physical calmness when the mind becomes focused [69]. Our results are therefore somewhat surprising and need further evaluation.

However, they could also be connected to the effects for the dimension mood, where moderate and high values for the variation of speed showed higher odds for the probability of positive changes compared to the lowest ones. Although both categories showed higher odds, the effect was significant for moderate values between 5 to 10 rpm compared to a variation of less than 5 rpm. These results, in turn, would also suggest that a certain degree of variation in the movement corresponds to the solving of different types of tasks, the participants do not have to focus a lot on, and as a consequence, the perception of mood might increase after the event of use. In contrast to the variation of speed, moderate and high values for the speed itself showed lower odds of reporting an improvement in mood compared to the lowest ones. Therefore completing less than 35 rpm while using the devices seems to be more pleasant compared to using them with a higher cadence. This might be related to perceiving lower exertion and effort while using the DOWs with this cadence, which could be rather connected to improvements in mood than a higher speed of using [57].

### Limitations and Strengths

The present study has some limitations. The objective intensity of using the Deskbikes (for example in watts) could not be measured due to the design of the device and the measurement system. Therefore revolutions per minute were registered and interpreted as the speed of pedaling but without a relationship with the actual intensity of using the cycling devices. Another aspect concerns the assessment of well-being using a modified version of the EZ-scale and interpreting the outcomes. The validity of the original questionnaire constructed by Nitsch is given by the use of judgement collectives and using binary structure analysis to define the scales and items [48]. As our study design required multiple repeated measures of the same construct, the original version containing 40 items was not feasible and was modified. By reducing the dimensions being represented by two bipolar arranged items, a dimensional analysis of reliability was not possible. Despite that, short versions of the EZ-scale are used to assess the concept of well-being in other repeated measures study designs [70,71].

Regarding the interpretation of the outcomes for the modified EZ-scale some aspects should be kept in mind: although using logistic GEE regression models enables to analyze every event of use with taking the correlation of repeated measurements on one subject into account, the results should not be interpreted as causal connections between characteristics of use and the dimensions of well-being. Especially as the categories are compared to one reference category but not to each other, assumptions about relationships between the characteristics of use and well-being should be made cautiously.

Additionally, some participants did not respond to the questionnaire until they returned the device to the borrowing station instead of responding immediately after each bout of use. Therefore, although deleting datasets where delays between answering the scale and using the device where obvious, some of the responses might include breaks with behavior occurring which affects short-term well-being as well.

Another important aspect to consider is, that the surveys were based on self-reported outcomes. This might put a burden on some participants as well as it might include answers from participants being potentially already motivated to rate positively. Both aspects can lead to a bias in the outcomes. Therefore, positive changes in the scores might not be due to the use of a cycling device in the first place. This is even more important as in the repeated measurement study design the participants served as their own “active” control group instead of implementing one group not using the Deskbikes. But as the study was conducted in a real-life office environment, no systematic effects were expected to occur when testing against working at the desk without cycling as the tasks completed could not be controlled.

Conducting the study in a real-life office environment is an important strength as the assessment of the characteristics of use in detail and to the extent in the present study are not common in field studies. The present work complements the existing findings of using innovative dynamic workstations in occupational settings. Additionally, the knowledge gained about the positive short-term effects on well-being and the exploration of relationships to characteristics of use contribute to limited scientific findings regarding the psychological effects of using dynamic workstations.

## 5. Conclusions

In summary, the results of the present study confirm previous findings regarding the use of dynamic workstations in real-life office environments. When choosing the frequency and duration of using the devices voluntarily, participants show characteristics of use which differ highly from each other. Half of the employees used the cycling devices regularly. For them, using the Deskbikes might even contribute to levels of light physical activity, considering an average duration of use of at least 20.1 min per event per day. Additionally, the analyses of the short-term changes of well-being while using the devices show that in general using the Deskbikes might be associated with changes of outcomes representing mental health. But in contrast to the linear relationship between different levels of physical activity and effects on the cardiovascular system, this seems to not apply to the effect on psychological parameters in the same way. There were mixed effects to be found for characteristics of use (duration of use per event, speed and consistency of using) for three dimensions of well-being (recovery, mood and calm), which show possible dose-response relationships. Some of these relationships display positive or negative linear correlations. Others display inverted u-shape relationships, as there were higher and lower odds at the same time to be found for different distributions (classes) within one characteristic of use compared to the reference class. As these results are inconclusive, more investigations are needed to further examine possible relationships of characteristics of use, especially regarding different distributions within these characteristics. Also, as age and gender seem to have an effect on the probability of reporting improvements, they should be examined in future work to extend the recommendations about using DOWS stratified for these characteristics. Moreover, interventions to motivate employees to use the Deskbikes especially when experiencing more negative mood states, should be tested. Such an approach would enable stronger effects to prevent possible stress-related health problems or a loss of productivity at the workplace. In conclusion, the implementation of Deskbikes in real-life office environments might be a way to contribute to workers’ psychological health and provide economical savings for companies at the same time.

## Figures and Tables

**Table 1:** Anthropometric information about the study sample; M = Mean; SD = Standard Deviation.

Participants	Number	Age in Years	Height in Meters	Weight in kg	BMI
		M (SD)	M (SD)	M (SD)	M (SD)
All	36	41.0 (10.7)	1.74 (0.10)	76.9 (18.3)	25.2 (5.0)
Male	17	43.2 (11.4)	1.82 (0.08)	86.4 (13.9)	26.0 (2.8)
Female	19	39.1 (10.1)	1.71 (0.05)	72.2 (18.0)	24.7 (6.4)

**Table 2:** Categories of characteristics of use; rpm = rounds per minute; SD = Standard Deviation.

Categories	Characteristics of Use			
	Duration of Use per Event (min)	Number of Use Bouts per Event	Mean rpm per Event	Mean SD of rpm per Event
1	≤22.1	1	≤35.35	≤5.44
2	22.1 to 36.13	1 to 4	35.35 to 40.40	5.44 to 10.38
3	36.13 to 51.83	≥4	≥40.40	≥10.38
4	≥51.83	-	-	-

**Table 3:** Minimum and maximum values of characteristics of use per event (817) for 36 participants.

Variables	Min	Max
Duration of use per event in min	1	151.7
Total number of use bouts per event	1	25
Mean revolution per minute (rpm) per event of use	1	46.4
Mean standard deviation of rpm per event of use	0	24.5

**Table 4:** Results of the paired *t*-test for pre- and post-measurements of each dimension of the modified EZ-scale for 33 participants. M = Mean, SD = Standard Deviation; * = significant effect (*p* ≤ 0.05).

Variables	Pre	Post	*t*-Test		
	M (SD)	M (SD)	*t*	*p*-Value	Effect Size (d)
recovery	4.50 (0.92)	4.77 (0.64)	−1.753	0.089	0.33
self-confidence	5.08 (0.87)	5.34 (0.59)	−2.441	0.020 *	0.32
calm	5.16 (0.80)	5.38 (0.54)	−2.991	0.005 *	0.28
mood	5.00 (0.80)	5.26 (0.61)	−3.867	0.001 *	0.34
willingness to perform	4.46 (0.91)	4.80 (0.64)	−3.094	0.004 *	0.40

**Table 5:** Results of the logistic Generalized Estimating Equation (GEE) regression on changes in short-term well-being. All models are adjusted for age, gender and relative activity; OR = odds ratio; 95%-CI = 95%-confidence interval; *p* = *p*-value; significant effect when *p* ≤ 0.05.

Parameter	Category	Recovery			Self-Confidence			Calm			Mood			Willingness to Perform		
		OR	95%-CI	*p*	OR	95%-CI	*p*	OR	95%-CI	*p*	OR	95%-CI	*p*	OR	95%-CI	*p*
Duration of use per event in min	≤22.1	1			1			1			1			1		
	22.1 to 36.13	1.76	0.90–3.42	0.10	0.88	0.57–1.35	0.55	1.44	0.73–2.83	0.30	1.07	0.47–2.45	0.87	1.18	0.72–1.94	0.52
	36.13 to 51.83	2.22	1.18–4.20	0.01	0.60	0.31–1.15	0.12	1.95	0.89–4.28	0.10	1.52	0.68–3.42	0.31	1.36	0.80–2.34	0.26
	≥51.83	2.21	0.88–5.53	0.09	1.09	0.50–2.38	0.84	1.21	0.32–4.62	0.78	1.00	0.41–2.46	1.00	1.38	0.73–2.59	0.32
Number of bouts of use per event	1	1			1			1			1			1		
	2 to 4	0.97	0.64–1.48	0.89	0.85	0.42–1.72	0.65	1.15	0.52–2.53	0.74	0.88	0.45–1.71	0.70	0.92	0.44–1.93	0.82
	≥4	0.59	0.20–1.71	0.33	0.69	0.10–4.66	0.70	1.26	0.41–3.90	0.69	0.91	0.30–2.76	0.87	1.81	0.60–5.43	0.29
Mean rpm per event of use	≤35.35	1			1			1			1			1		
	35.35 to 40.40	1.60	1.10–2.33	0.01	0.85	0.56–1.29	0.45	0.77	0.54–1.08	0.13	0.50	0.32–0.78	≤0.01	0.99	0.59–1.66	0.96
	≥40.40	1.03	0.63–1.69	0.91	0.75	0.42–1.31	0.31	1.02	0.43–2.42	0.96	0.65	0.39–1.08	0.10	1.06	0.51–2.22	0.87
Mean SD of rpm per event of use	≤5.44	1			1			1			1			1		
	5.44 to 10.38	1.34	0.93–1.92	0.12	1.50	1.00–2.27	0.052	1.46	0.76–2.77	0.25	1.61	1.03–2.52	0.04	0.92	0.61–1.38	0.68
	≥10.38	0.52	0.31–0.86	0.01	0.71	0.37–1.36	0.30	1.82	1.22–2.73	≤0.01	1.12	0.57–2.20	0.73	0.59	0.35–1.00	0.051
